# A Case Report of Successful Live Pregnancy Following Embryo Transfer in a Thin Endometrium

**DOI:** 10.7759/cureus.66363

**Published:** 2024-08-07

**Authors:** Jijisha Ali, Shazia Magray, Eman Ahmed, Sami Talo

**Affiliations:** 1 Obstetrics and Gynaecology, Mediclinic Welcare Hospital, Mediclinic Middle East, Dubai, ARE; 2 Reproductive Medicine and Infertility, Bourn Hall Fertility Centre, Mediclinic Middle East, Dubai, ARE; 3 Clinical Embryology, Bourn Hall Fertility Centre, Mediclinic Middle East, Dubai, ARE; 4 College of Medicine, Mohammed Bin Rashid University of Medicine and Health Sciences, Dubai, ARE

**Keywords:** assisted reproductive technologies, infertility, frozen embryo transfer, recurrent, thin endometrium

## Abstract

Managing a thin endometrium is a common challenge in assisted reproductive treatments. The thickness of the endometrium is crucial for embryo implantation, with younger patients generally having higher success rates even with a thinner lining. A frozen embryo transfer cycle often allows for a more thorough assessment of the endometrium compared to a fresh transfer. We present a case of a 36-year-old woman who presented to our fertility center with primary infertility for six years. Despite having regular menstrual cycles, her endometrial thickness consistently measured between 5 and 6.0 mm on ultrasonography. She underwent ovarian stimulation using an antagonist protocol, resulting in the retrieval of oocytes and the freezing of three embryos. However, three frozen embryo transfer cycles were cancelled due to inadequate endometrial thickness (ranging from 4.3 to 5.2 mm). In the fourth cycle, she was treated with gonadotropins with the goal of achieving two to three follicles and improved endometrial thickness. Triggering was performed on day 15, followed by the transfer of one frozen embryo at an endometrial thickness of 5.7 mm. Her beta-human chorionic gonadotropin (hCG) level was positive, with an initial value of 136.9 mIU/mL, and she subsequently delivered a healthy baby. This case highlights the challenges of managing a thin endometrium in assisted reproductive techniques. Through persistent efforts and tailored treatment protocols, a successful live birth was achieved despite recurrent thin endometrium. This case underscores the importance of individualized treatment strategies in overcoming endometrial challenges in infertility treatments.

## Introduction

The effectiveness of assisted reproductive technologies (ARTs) largely relies on the implantation of a genetically healthy embryo into a receptive endometrium [1]. It is suggested that two-thirds of implantation failures are due to poor endometrial receptivity and disrupted communication between the embryo and the endometrium [2]. Pregnancy rates tend to rise as endometrial thickness (EMT) increases from 8 to 14 mm [3]. An EMT of less than 7 mm during the implantation window (typically between days 19 and 23 of the menstrual cycle), as observed on ultrasound, is generally classified as a thin endometrium.

Research indicates that embryo implantation and live birth rates improve when the EMT exceeds 8 mm on the day of trigger shot administration [4]. However, the minimum EMT associated with a successful full-term live birth in assisted reproduction is reported to be 3.5 mm [5]. The endometrium should be assessed using a transvaginal probe with the bladder empty. This method places the transducer closer to the endometrium and employs a higher frequency (≥5 to 8 MHz). This results in enhanced resolution and visualization, though it reduces penetration depth. The EMT should be measured in the sagittal plane or long axis, taking the measurement from the thickest echogenic area from one stratum basalis interface across the endometrial canal to the opposite stratum basalis interface. It is important not to include the surrounding inner myometrial lucency in this measurement [6].

A persistently thin endometrium is often linked to past extensive curettage, infections, Asherman's syndrome, or uterine irradiation [7]. Efforts to enhance overall endometrial growth and pattern have not yet been validated. Various treatments have been attempted, including operative hysteroscopy, intrauterine infusion of granulocyte colony-stimulating factor (G-CSF), and methods to improve endometrial blood supply such as aspirin, vitamin E, pentoxifylline, L-arginine, or sildenafil. Regenerative medicine approaches have also been explored, and certain studies have suggested that intrauterine platelet-rich plasma (PRP) can enhance EMT [8]. However, none of these methods have been shown to consistently improve cases of thin endometrium.

## Case presentation

A 36-year-old woman with a six-year history of primary infertility presented to our fertility center. She reported regular menstrual cycles and had previously undergone two laparoscopic surgeries for Grade IV endometriosis. Her anti-Müllerian hormone (AMH) level was measured at 0.5 ng/mL (reference range: 1.0-4.0 ng/mL), indicating diminished ovarian reserve. The rest of her endocrine profile was within normal limits, except for an elevated prolactin level. Her husband's semen analysis was normal. Ultrasonography performed on day 2 of her menstrual cycle revealed five antral follicles in the right ovary and three in the left ovary. The EMT on day 2 was 4.8 mm (reference range: 7-8 mm). She was prescribed cabergoline 0.25 mg weekly and planned for a short antagonist cycle of ART.

She commenced treatment with follitropin alfa 300 IU daily starting from day 2 of the menstrual cycle, as shown in Table 1. On day 7 of the ART cycle, cetrorelix acetate 0.25 mg was added to prevent premature ovulation. On day 10, ultrasonography showed an EMT of 6 mm. The patient was then given menotropins (a combination of follicle-stimulating hormone (FSH) 225 IU and luteinizing hormone (LH) 225 IU), and cetrorelix acetate was continued. On day 12, ultrasonography showed a very thin endometrial lining of 5.5 mm (reference range: 7-8 mm). Triggering was performed on the same night, and egg retrieval occurred 36 hours later. Three embryos of grade AB were successfully frozen (Tables 1, 2).

**Table 1:** Folliculogram showing the short antagonistic cycle of ART. OPU: oocyte pick-up; ART: assisted reproductive technology; EMT: endometrial thickness; FSH: follicle-stimulating hormone; LH: luteinizing hormone.

OPU cycle 1	Day 2	Day 7	Day 9	Day 12	DAY 14
EMT (reference range: 7-8 mm)	4.8 mm	5 mm	6.0 mm	5.5 mm	5.8 mm
Right ovary: follicle number	Three antral follicles	Four follicles	Three follicles	Five follicles	OPU
Right ovary: follicle size	2-3 mm	10-12 mm	16-13 mm	14-20 mm	
Left ovary: follicle number	Five antral follicles	Five follicles	Four follicles	Five follicles	
Left ovary: follicle size	2-3mm	10-12 mm	13-16 mm	14-17 mm	
Gonal-F (follitropin alfa)	Started 300 IU	300 IU to continue	Stopped Gonal-F and started menotropins	Stopped menotropins	
Cetrorelix acetate	Was on Gonal F only	0.25 mg added to continue	0.25 mg to continue	0.25 mg last dose	
Menotropins (FSH 225 IU and LH 225 IU)	Was on Gonal F only		225 IU started to continue till Day 11	Triggering done with choriogonadotropin alfa injection (Ovitrelle 250 mcg equivalent to 6500 IU) at night	

**Table 2:** Folliculogram showing the FET cycle. EMT: endometrial thickness; FET: frozen embryo transfer; FSH: follicle-stimulating hormone; LH: luteinizing hormone.

FET cycle 1	Day 3	Day 12	Day 15	Day 22
EMT (reference range: 7-8 mm)	5.2 mm	5.5 mm	5.5 mm	5.7 mm
Cetrorelix acetate	Was on menotropins 75 IU only	0.25 mg started	0.25 mg last dose	FET done
Menotropins (FSH 75 IU and LH 75 IU)	75 IU started and to continue	75 IU to continue till Day 14	Triggering done with choriogonadotropin alfa injection (Ovitrelle 250 mcg) at night.	

**Figure 1:**
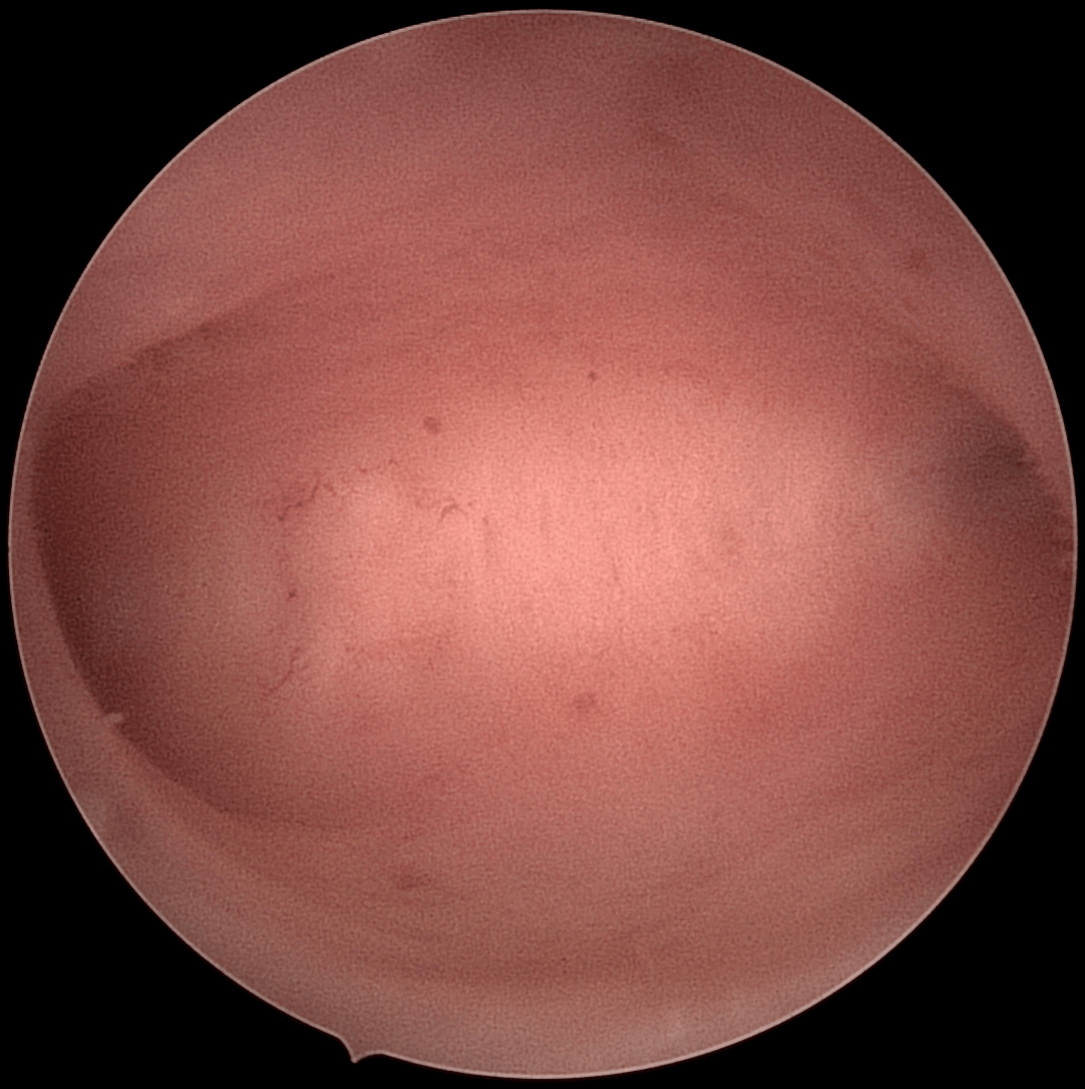
Hysteroscopic image revealing a normal endometrium in the patient.

For the subsequent frozen embryo transfer (FET) cycle, the patient was started on estradiol valerate 4 mg three times daily, both orally and vaginally, due to thin endometrium noted on day 2. However, by day 9, the endometrial lining was only 4.3 mm (reference range: 7-8 mm), with fluid present in the uterine cavity. The cycle was cancelled due to persistent thin endometrium and intrauterine fluid. A hysteroscopy was performed and revealed a normal endometrium, as shown in Figure 1. Similar ultrasound findings were seen after three months, and the patient was started on estrogen patches (100 mcg every three days) and letrozole 5 mg for seven days. This cycle was also cancelled due to persistent intrauterine fluid and thin endometrium.

**Figure 2:**
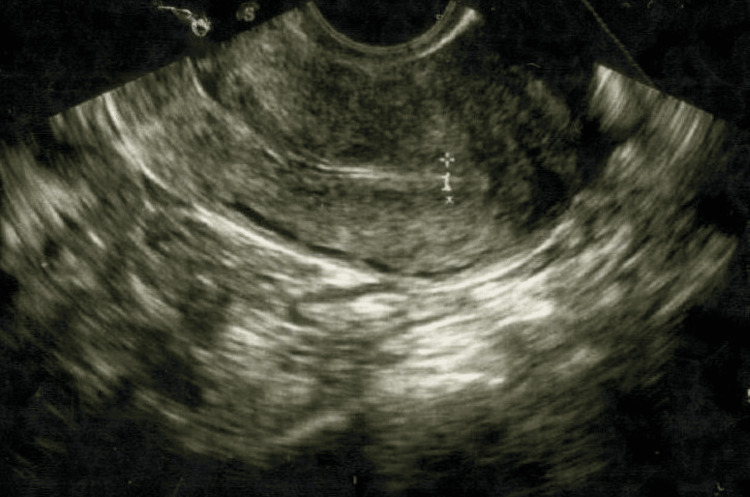
Ultrasound showing the EMT of the patient at the time of embryo transfer. EMT: endometrial thickness.

In the subsequent cycle, the patient was administered gonadotropins (FSH 75 IU and LH 75 IU) from day 3. Cetrotelix acetate 0.25 mg was started on day 12. By day 12, the EMT was 5.5 mm (reference range: 7-8 mm). Choriogonadotropin alfa was administered on day 15, followed by single embryo transfer of grade AB on day 22, with an EMT of 5.7 mm (reference range: 7-8 mm) at the time of transfer (Figure 2). The patient was started on estradiol valerate 2 mg twice daily and progesterone soft gel capsules twice daily. Beta-human chorionic gonadotropin (hCG) measured on day 31 was positive, with a value of 136.9 mIU/mL (reference range: <5 mIU/mL). She subsequently delivered a healthy female baby weighing 3.1 kg at 37 weeks of gestation.

## Discussion

**Figure 3:**
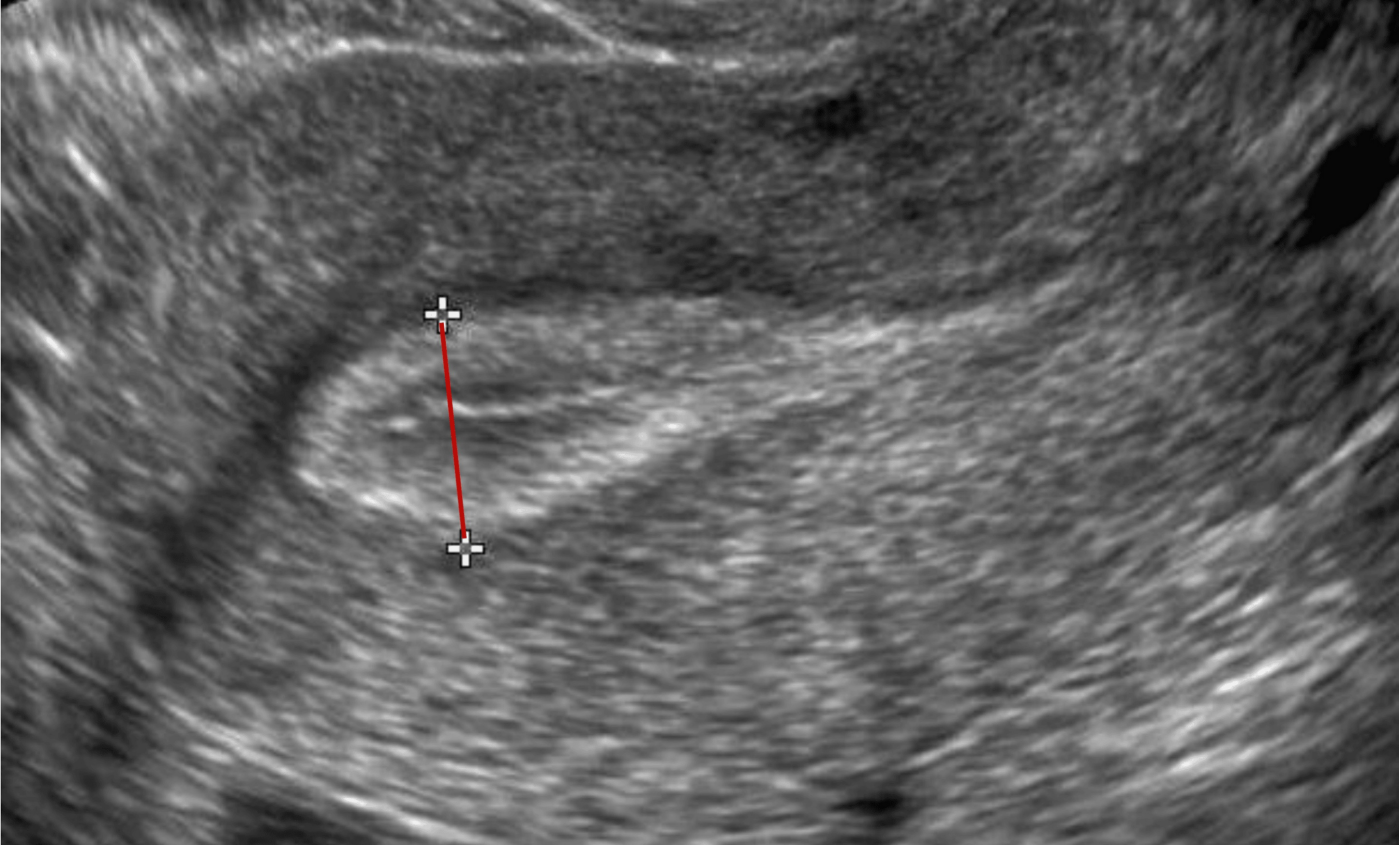
Ultrasound showing the measurement technique for EMT. EMT: endomterial thickness.

Managing a thin endometrium is a frequent challenge for patients undergoing assisted reproduction. Clinical practice typically involves measuring EMT. Despite conflicting study results and a lack of consensus on the correlation between EMT and IVF outcomes, an EMT of less than 7 mm is generally considered unfavorable. The EMT is measured in the sagittal plane or long axis, taking the measurement from the thickest echogenic area from one stratum basalis interface across the endometrial canal to the opposite stratum basalis interface, as shown in Figure 3.

Recent studies indicate that EMT on the day of hCG trigger can influence pregnancy outcomes, with a thin endometrium posing a clinical challenge [9]. To address this, various strategies have been employed in clinical practice to enhance endometrial growth. These strategies include the administration of estrogen, low-dose aspirin, vitamin E, vaginal sildenafil citrate, and intrauterine perfusion with granulocyte colony-stimulating factor (G-CSF). These methods have not shown consistent improvement in EMT. For example, a 2024 case report by Huang et al. noted that EMT did not significantly increase with the use of aspirin. However, the study documented a successful pregnancy with an EMT of 3.8 mm [10]. This suggests that even with a thin endometrium, appropriate interventions can lead to successful pregnancies. Agrawal et al. reported another case in 2022 where a successful pregnancy was achieved in a patient with a persistently thin endometrium [11]. For patients with a thin endometrium, it is important to focus not only on increasing thickness but also on improving endometrial blood supply and morphology to enhance pregnancy rates [10].

In a 2013 study by Chen and Chen, three patients who previously failed to develop an EMT of at least 6 mm over at least three cycles, including natural cycles, extended estrogen treatment cycles with aspirin, or letrozole stimulation cycles, achieved significant endometrial growth and successfully conceived following the administration of 20 mg of tamoxifen daily for five days [12].

In our case report, we achieved successful pregnancy with an EMT of 5.7 mm (reference range: 7-8 mm). Our patient was stimulated with gonadotropins for 14 days, followed by trigger with choriogonadotropin alfa 250 mcg on the 15th day. Her EMT remained in the range of 5-6.0 mm. Clinicians often change stimulation medications when faced with a thin endometrium. A meta-analysis by Weiss et al. found that clomiphene and letrozole were both linked to a thinner endometrium compared to gonadotropins in ovarian stimulation cycles [13]. When a clinician encounters a persistently thin endometrium, it is important to evaluate the patient's condition and preferences. Clinicians should make every effort to employ a variety of methods to address the issue. It is important to note that for patients with a thin endometrium, the potential to achieve a thicker endometrium in future ovarian stimulation cycles is uncertain.

Furthermore, in cases of patients with a thin endometrium, it is recommended to opt for a frozen cycle instead of a fresh transfer. This approach allows for a more detailed assessment of the endometrium and offers the advantage of postponing the transfer if the lining has not developed adequately [14]. Moreover, for women over 35 years of age, FETs have shown significantly higher rates of biochemical pregnancy, clinical pregnancy, and live births compared to fresh embryo transfers [15].

## Conclusions

This case highlights the challenges and successful outcomes of assisted reproductive technology (ART) in a patient with primary infertility and severe endometriosis. Despite multiple cycles complicated by a persistently thin endometrium and intrauterine fluid, the use of gonadotropins, estrogen therapy, and careful monitoring resulted in a successful pregnancy and delivery. It is important to conduct an endometrial assessment prior to the index cycle in such cases, as young patients may still have a reasonable chance of implantation even with a thin endometrium. For those preparing for a FET, the status of the endometrium during the stimulation cycle should be carefully considered. Physicians must weigh the potential outcomes of proceeding with treatment against the possibility of exploring alternative approaches for patients with a thin endometrium. Currently, there is limited evidence supporting specific protocols or adjuvants that can significantly enhance pregnancy outcomes for these patients. Further research is needed to establish effective strategies for managing thin endometrium in assisted reproduction.
